# Deciphering the biology of NKG2C+ natural killer cells

**DOI:** 10.18632/oncotarget.4468

**Published:** 2015-06-14

**Authors:** Alexander Rölle

**Affiliations:** Department of Tumor Immunology, German Cancer Research Center (DKFZ), Heidelberg, Germany

**Keywords:** NK cells, HCMV, HLA-E, NKG2C, IL-12

The classic division of the immune system into an innate and an adaptive branch has been one of the dominant paradigms for immunologists shaping both questions and interpretations in the field for many years.

This traditional concept has been challenged recently and the once clear boundaries are beginning to blur. Exciting new findings demonstrate that during the course of an immune response, Natural Killer (NK) cells, generally considered innate immune cells, can display certain features of adaptive immunity such as subset expansion, increased longevity and more potent effector functions upon secondary challenge.

Roughly a decade ago, a first report [1] described a lasting imprint of human cytomegalovirus (HCMV) infection on the human NK cell repertoire, highlighting the expansion of an NK cell subset carrying the activating receptor NKG2C. This finding was surprising, given that the conventional view of innate immunity would not entail any long-lasting alterations after a primary response. In subsequent years, several pioneering studies in mice suggested the existence of NK cell memory or NK cells with memory-like features [2, 3]. These data sparked renewed interest in the expansion of NKG2C+ NK cells in humans.

After the initial description by Guma and co-workers, the expansion of NKG2C+ NK cells was also observed in other infections, often in association with CMV-seropositivity [4]. The role of HCMV as a pivotal driver for subset expansion was further substantiated by several studies focusing on the dynamics of the NK cell compartment during active HCMV disease/reactivation after transplantation a clinical context in which HCMV represents a major and frequent complication. However, in spite of the growing body of literature describing the expansion of NKG2C+ NK cells in a broad range of systems, the underlying molecular mechanisms remained largely unclear.

In our recent study in the *Journal of Clinical Investigation* we set out to elucidate the molecular determinants driving the expansion of NKG2C+ NK cells in HCMV infection [5]. In a co-culture system we noticed expansion of NKG2C+ NK cells in response to HCMV-infected fibroblasts when accessory cells contained in PBMC were present but not when purified NK cells were exposed to infected fibroblasts. After switching to a transwell system, we could show that cell-to-cell contact between PBMCs and NK cells was not essential for the expansion of NKG2C+ NK cells, which suggested the involvement of soluble factors. After neutralization of different cytokines or blockade of their respective receptors we identified IL-12 as a key cytokine for subset expansion. Interestingly, neutralization of IL-12 also largely abolished the upregulation of CD25 on NK cells in infected co-cultures. CD25 is required for the formation of the high-affinity variant of the IL-2 receptor, which is presumably a precondition for efficient expansion of NKG2C+ NK cells.

We then identified CD14+ monocytes as an important source for IL-12 in our system. The critical role of CD14+ monocytes for NK cell survival and subset expansion was confirmed in depletion experiments but not solely limited to the production of IL-12. Accordingly, we could reconstitute expansion of NKG2C+ NK cells in response to infection in purified NK cell cultures after supplementing them with CD14+ monocytes. Finally, we demonstrated that the upregulation of HLA-E (the cellular ligand for CD94/NKG2C) on infected cells was strictly required for the initation of subset expansion. The crosstalk between NK cells and monocytes - partially mediated by IL-12 - in combination with increased HLA-E levels on potential target cells might define unifying features for the expansion of NKG2C+ NK cells in different systems [5].

Fascinating new studies describe another subset of NK cells that is observed in about one third of all individuals and which is characterized by a deficiency for the adaptor protein FcεRγ [6, 7]. This population is not identical but seems to largely overlap with expanded NKG2C+ NK cells. Both subsets display functional superiority, in particular in terms of mediating antibody-dependent cellular cytotoxicity (ADCC) and cytokine responses.

**Figure:**
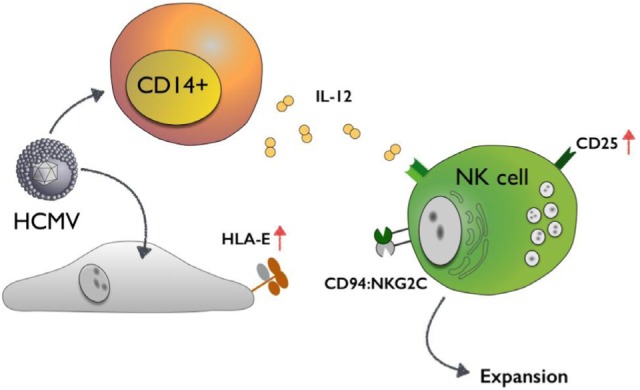
Human Cytomegalovirus (HCMV) induces HLA-E on infected cells and triggers CD14+ monocytes to secrete IL-12, which subsequently leads to the upregulation of CD25 on Natural Killer (NK) cells. In concert, these factors drive the expansion of an NK cell subset carrying the activating receptor CD94/NKG2C.

Translational approaches aiming to utilize NK cells in tumor therapy might greatly benefit from targeting these novel “adaptive” subsets. A more detailed analysis of their biology will undoubtedly be a rewarding field of investigation in the future.

## References

[1] Guma M (2004). Blood.

[2] Sun J (2010). Immunol Rev.

[3] Paust S (201). Nat Immunol.

[4] Rölle A (2013). PLoS Pathog.

[5] Rölle A (2014). J Clin Invest.

[6] Schlums H (2015). Immunity.

[7] Lee J (2015). Immunity.

